# Vivisections—Letters from Henry Bergh and Dr. Dalton

**Published:** 1866-11

**Authors:** 


### Vivisections—Letters from Henry Bergh and Dr. Dalton.

On the 27th of September last, Mr. Henry Bergh, President of the Society for the Prevention of Cruelty to Animals, addressed a letter to Dr. Edward Delafield, President of the College of Physicians and Surgeons in New York, asking his opinion "whether the practice of that branch of surgery known as vivisection could not be wholly dispensed with, or so modified, without detriment to science, as to exempt from suffering, by the employment of anæsthetics, the animals operated upon."

Dr. Delafield referred Mr. Bergh's letter to Dr. Dalton, professor of physiology in the college. On the 22d of September Dr. Dalton replied that the practice of vivisection had been conducted in that college "in conformity with the most humane methods known to the profession," but added that "suffering is prevented in nearly all instances by the previous use of anæsthetic agents, in the same manner as in surgical operations on the human subject. The cases in which they cannot be used for this purpose are very rare."

Mr. Bergh rejoined, under date of October 6, acknowledging the receipt of Dr. Delafield's reply, and the inclosure from Dr. Dalton, and remarking: "I cannot overlook the avowal made by Dr. Dalton, that 'there are cases in which anæsthetics cannot be used, although rare.'" He added a request for a specification of the cases in which anæsthetics could not be used, and the reasons for those exceptions. Dr. Dalton replied to this request as follows:

* * * "Inswer to your request, I would say that the cases referred to, where anæsthetics cannot be used, are those in which the insensibility produced by anæsthetics would interfere with the performance of the experiment and defeat its objects—as in those of Magendie on the nervous system, in which the object of the experiment was to ascertain the presence or absence of sensibility in particular parts. The special character of these cases will undoubtedly vary in course of time with the progress of medical inquiry.

"Yours, respectfully,
"J. C. DALTON,
"Professor of Physiology."

Mr. Bergh makes a lengthy reply to this letter charging inhumanity and barbarity, and quoting letters from various surgeons sustaining his position.

Dr. J. C. Dalton's reply to Mr. Bergh shows that the sole and only ultimate object of this practice is the relief of human suffering and the cure of human diseases; that it is not cruel but humane and unobjectionable, being almost always done while the animals are under influence of ether or chloroform. He says:—"It has always been believed that the fullest instruction in physiology, as well as in the elementary branches, is essential to the due preparation of intelligent and successful physicians. The more complete and efficient this instruction is made, the more competent will be the practitioners who every year are sent out to join the ranks of the medical profession. This instruction is made as complete as possible by ocular demonstrations of many important facts, but this is always done, as I believe, in a reasonable and proper way."

